# The role of the lower extremity functional scale in predicting surgical outcomes for total joint arthroplasty patients

**DOI:** 10.1186/s42836-021-00106-3

**Published:** 2022-02-01

**Authors:** Justin J. Turcotte, McKayla E. Kelly, Alyssa B. Fenn, Jennifer J. Grover, Christina A. Wu, James H. MacDonald

**Affiliations:** Anne Arundel Medical Center, 2000 Medical Parkway, Suite 503, Annapolis, MD 21401 USA

**Keywords:** Total knee arthroplasty, TKA, Total hip arthroplasty, THA, Lower extremity functional scale, LEFS, Outcomes, Predictive model

## Abstract

**Background:**

The purpose of this study was to evaluate the relationship between lower extremity functional scale (LEFS) scores with postoperative functional outcomes for total joint arthroplasty (TJA) patients and to investigate the utility of this tool to create an individualized plan of care perioperatively.

**Methods:**

Patients undergoing primary TJA at a single institution from 2016 to 2019 was retrospectively reviewed by a univariate analysis in terms of patient characteristics and outcomes across LEFS quartiles. Multivariate regression models were constructed to evaluate the association between the LEFS quartile and outcomes after controlling for confounding factors.

**Results:**

A total of 1389 patients were included. All patients had a documented LEFS pre- and postoperatively with the last value documented at least 60 days to a maximum of 1 year after surgery. The following cutoffs for LEFS quartiles were observed: quartile 1 preoperative LEFS ≤27, quartile 2 ranges from 28 to 35, quartile 3 ranges from 36 to 43, and quartile 4 ≥ 44. Patients with a higher comorbidity burden and ASA score were more likely to have a lower LEFS. Higher levels of preoperative function were significantly associated with shorter LOS and higher rates of same day discharge, independent ambulation, mobility and activity scores, and rates of discharge home.

**Conclusion:**

These findings suggest that LEFS is a useful tool for aiding clinical resource allocation decisions, and incorporation of the measure into existing predictive models may improve their accuracy.

**Supplementary Information:**

The online version contains supplementary material available at 10.1186/s42836-021-00106-3.

## Background

Osteoarthritis (OA) is one of the most disabling conditions associated with ageing. With an estimated 40% of people over the age of 65 afflicted by OA, the prevalence of this degenerative condition warrants further investigation into the best course of treatment for these patients. Hip and knee OA specifically are associated with increased pain and disability which can profoundly affect a patient’s physical, psychological, and socioeconomic well-being [1]. Total joint arthroplasty (TJA) is often required in the cases of severe OA and has emerged in recent years as one of the most commonly performed procedures in the United States [1–3]. Therefore, understanding the relationships between physical function, surgical setting, and the length of stay (LOS) is crucial to improving patient care [1, 4]. Patient factors relating to prior level of function have been found to aid surgeons in determining surgical setting (ambulatory surgery center or hospital). Within previously described predictive models, preoperative levels of physical function have been inversely correlated with hospital LOS [5, 6].

Physical function is traditionally evaluated using patient-reported measures collected during the pre- and postoperative periods. Previously investigated functional scales include the Western Ontario and McMaster Universities Osteoarthritis Index (WOMAC), Short Form-36 (SF-36), Hip Disability and Osteoarthritis Outcome Score (HOOS), and EuroQol 5D (EQ-5D). WOMAC includes basic questions about pain, symptoms, and daily activity for hip osteoarthritis patients. The HOOS includes all questions from the WOMAC in addition to sub-scales for recreation and sport activities [7, 8]. The EQ-5D provides a single value to evaluate health status mobility, self-care, usual activities, pain/discomfort, and anxiety/depression [9]. The EQ-5D provides a simple yet descriptive profile and single index value for health status that can be used in the clinical and economic evaluation of healthcare. Similarly, the SF-36 measures functional status, well-being, and overall health with eight sub-scales [10].

Although the relationship between LOS and patient-specific factors has been well studied, there is not a standard tool able to accurately predict postoperative outcomes for TJA patients [11, 12]. The lower extremity function scale (LEFS) is a valid and reliable tool that has been used in orthopedic physical therapy (PT) settings since it was first described in 1999 by Binkley* et al* [13]. LEFS is a 20-item condition-specific questionnaire applicable to patients with musculoskeletal conditions of the lower extremities (A copy of the LEFS instrument is presented in Additional file 1). The LEFS was first presented by Binkley *et al* [13] as a patient-reported measure of functional status for those with a lower extremity musculoskeletal problem. The scale is meant to serve two purposes: (1) document physical therapy outcomes for multiple patients for quality assurance or research purposes and (2) document the functional status of individual patients to allow for accurate goal setting and measurement of progress. The 20 items included in the LEFS each are scored from 0 (extreme difficulty/incapable of performing task) to 4 (no difficulty performing task). The sum of these items demonstrates the functional status of the patient. A maximum score of 80 indicates no limitations and a minimum score of 0 is indicative of extreme functional limitations [13, 14]. During original validation of the LEFS, the test-retest reliability of LEFS scores was excellent (*R* = 0.94 [95% lower limit confidence interval (CI) = 0.89]). When compared to the SF-36 physical function score, the correlation was high (*R* = 0.80 [95% lower limit confidence interval (CI) = 0.73]), and the sensitivity to change of the LEFS was superior to that of the SF-36 in the total joint population [13]. Mehta *et al* [14] conducted a systematic literature review of 27 articles analyzing the reliability, validity, and sensitivity to change of LEFS. The scale again demonstrated excellent test-retest reliability. The responsiveness of the LEFS scores was excellent as suggested by consistently high effect size for various lower extremity conditions. Minimal detectable change at 90% confidence interval (MDC90) for the LEFS scores varied between 8.1–15.3. Pooled estimate of the MDC90 was 6 points and the minimal clinically important difference was 9 points in patients with lower extremity musculoskeletal conditions which are indicative of true change and clinically meaningful change, respectively. While the LEFS is a strong predictor of pain, there is little to no research investigating the possible relationship between LEFS score and orthopedic surgical outcomes, such as LOS and discharge disposition [13–15].

The purpose of this study was to evaluate the relationship between preoperative physical function, documented via the LEFS, and surgical outcomes for TJA patients. In addition, the study aimed to use LEFS to develop a predictive tool to streamline perioperative patient care and ultimately be used by clinicians to guide preoperative planning by identifying patients that may require more resource allocation. Additionally, preoperative identification of prior level of function in terms of LEFS scores may help determine appropriateness of surgical location and postoperative disposition.

## Methods

### Study population

This study was deemed institutional review board exempt by the institution’s clinical research committee. A retrospective review of patients undergoing primary hip or knee arthroplasty at a single institution from 2016 to 2019 was performed using a convenience sampling methodology. Revision surgeries were excluded from this study. A total of 1389 patients were included. All patients had a documented LEFS within 1 year preoperatively. Preoperative LEFS was defined as the last value documented within 1 year of surgery. These scores were then stratified by quartile for the patient population. Postoperative LEFS was defined as the last value documented at least 60 days to a maximum of 1 year after surgery.

### Perioperative protocol

All patients were subjected to the same perioperative protocols in a coordinated Joint Replacement Center Program, as described in a previous report by our institution [16]. Preoperatively, patients attended an education class for them and their caregivers, received written educational materials, were given a medical evaluation, and participated in a formal physical therapy with LEFS performed pre- and postoperatively and documented in the electronic medical record. Patients who were determined to benefit from additional support were given in-home physical therapy. Patients in our institution had the benefit of participating in an established, standard rapid recovery pathway for TJA patients which includes a multimodal pain management regimen with celecoxib, acetaminophen, pregabalin, and short-acting opioids. Patient-controlled analgesia and femoral nerve blocks were not used; anesthesia was either general or neuraxial as determined by an anesthesiologist in consultation with the patient and surgeon. Adductor canal blocks (ACB) were used for patients undergoing TKA at the discretion of the surgeon and the anesthesiologist. Patients also received tranexamic acid, and engaged in the-day-of-surgery ambulation when appropriate. Aspirin at 81 mg twice daily as the primary pharmacologic deep vein thrombosis prophylaxis with warfarin or apixaban was used in select high-risk patients. Prior to discharge, all patients achieved adequate pain control using oral medication, had stable vital signs, were able to safely ambulate and void. Final discharge clearance included consensus from the surgical, medical, and therapy providers. Postoperatively, all patients participated in a formal physical therapy with the type and setting determined on the basis of functional and social factors and consistent with established rapid recovery protocols.

### Data collection and statistical analysis

All data were extracted from an administrative database and from the electronic medical record via a structured query language. Manual chart review was not performed other than for data quality assurance. Patient demographics, comorbidities, and surgery details were collected. Comorbidities were defined by International Classification of Disease 10th Edition (ICD-10) codes present in the patients’ chart at the time of surgery. Hospital outcomes included the length of stay measured in days and hours, same day discharge, and discharge disposition (defined as home or post-acute facility). For immediate postoperative evaluation, therapy-specific outcomes included the last documented Activity Measure for Post-Acute Care (AM-PAC) 6-Clicks mobility and activity scores prior to discharge, and whether the patient ambulated independently. For the purpose of this study, independent ambulation was defined as contact guard assistance, supervision, modified independence or independence. The 6-Clicks mobility assessment is a validated assessment of acute care basic mobility [17–19]. All LEFSs were captured by a therapist in the ambulatory PT setting. The 20 items documented and the associated scoring are presented in Additional file 1. Descriptive statistics were performed, and univariate comparisons of patient characteristics and outcomes were performed across LEFS quartiles. One-way analysis of variance (ANOVA) was used for continuous endpoints and chi-square tests were conducted for categorical endpoints. Multivariate linear and logistic regression models were then constructed to evaluate the association between LEFS quartiles and outcomes after controlling for confounding factors. Variables that were found to be statistically significantly different across LEFS quartiles on the univariate analysis were entered into the models as control variables. American Society of Anesthesiologists (ASA) score was used as a proxy for severity of illness rather than including individual comorbidities. Simple linear regression was then performed to evaluate the relationship between hospital outcomes and postoperative LEFS.

## Results

Patients had an average age of 67.1 ± 9.4 years, and an average BMI of 30.5 ± kg/m^2^, with 58.7% of them being female and 82.9% of white race. The most common comorbidities in the population were primary HTN (56.5% of patients), GERD (32.3%), and anxiety or depression (22.8%); 38.9% of patients had a multiple comorbidity burden as quantified by an ASA score ≥ 3. TKA was performed on 58.0% of patients, and 63.4% received spinal anesthesia. Overall, the average length of stay was 1.4 ± 1.0 days, with 5.5% of patients being discharged on the day of surgery. The majority of patients (91.4%) were discharged home. The average preoperative LEFS was 36.3 ± 13.2 and the average postoperative LEFS was 52.2 ± 12.6 (Table 1).

**Table 1:** Population demographics and outcomes

Variable	*n* (%) or Avg. ± SD
All Patients	1389 (100.0)
Demographics	
Age – yrs.	67.1 ± 9.4
BMI- kg/m^2^	30.5 ± 5.5
Female	815 (58.7)
White Race	1151 (82.9)
Comorbidities	
Diabetes Mellitus	239 (17.2)
Sleep Apnea	237 (17.1)
COPD	58 (4.2)
Asthma	154 (11.1)
Liver Disease	17 (1.2)
AFIB	104 (7.5)
CHF	31 (2.2)
CAD	153 (11.0)
ESRD or CKD	91 (6.6)
GERD	448 (32.3)
Anxiety or Depression	317 (22.8)
Primary HTN	785 (56.5)
Surgery Information	
ASA ≥ 3	541 (38.9)
Spinal Anesthesia	880 (63.4)
THA	583 (42.0)
TKA	806 (58.0)
Hospital Outcomes	
LOS days	1.4 ± 1.0
LOS hours	39.4 ± 23.3
Same Day Discharge	77 (5.5)
Discharged Home	1269 (91.4)
Independent Ambulation^a^	810 (97.7)
Last Six Clicks Mobility Score^a^	18.9 ± 2.9
Last Six Clicks Activity Score^a^	22.4 ± 2.1
LEFS	
Preoperative	36.3 ± 13.2
Days from Preop. LEFS to Surgery	39.1 ± 55.1
Postoperative^ab^	52.2 ± 12.6
Days from Surgery to Postop. LEFS	110.3 ± 63.0

# of patients missing data:

Hospital ambulation – 579

Six Clicks Mobility – 413

Six Clicks Activity – 969

Postop. LEFS – 899

^a^Denotes missing data

^b^Postop LEFS defined as last LEFS from ≥60 days to ≤365 days from date of surgery

The following cutoffs for LEFS quartiles were observed: quartile 1 was a preoperative LEFS ≤27, quartile 2 from 28 to 35, quartile 3 from 36 to 43, and quartile 4 ≥ 44. When stratifying the development sample by quartiles, significant differences in body mass index (BMI), female gender, white race, diabetes mellitus, GERD, anxiety or depression, ASA score, spinal anesthesia, and procedure performed were observed (all *P* < 0.05). Notable trends included higher BMIs, and a greater proportion of females and non-white race in patients with lower levels of physical function as measured by the LEFS. Similarly, patients with higher comorbidity burden as measured by rates of individual comorbidities and ASA score were more likely to have a lower LEFS. These patients were less likely to receive spinal anesthesia (Table 2).

**Table 2:** Patient demographics, comorbidities, and surgery details by preoperative LEFS quartile

Variable	LEFS quartile 1 (≤ 27) *n* = 350	LEFS quartile 2 (28 to 35) *n* = 321	LEFS quartile 3 (36 to 44) *n* = 354	LEFS quartile 4 (> 44) *n* = 364	*P*-Value
Preoperative LEFS – avg. ± SD	20.0 ± 5.6	31.4 ± 2.3	39.6 ± 2.5	53.1 ± 7.1	**< 0.001**
Demographics					
Age – avg. yrs. ± SD	67.5 ± 10.2	67.0 ± 9.1	66.4 ± 9.2	67.5 ± 9.0	0.349
BMI- avg. kg/m^2^ ± SD	31.5 ± 5.9	30.8 ± 5.3	30.3 ± 5.5	29.3 ± 5.0	**< 0.001**
Female – *n* (%)	233 (66.6)	204 (63.6)	205 (57.9)	173 (47.5)	**< 0.001**
White Race – *n* (%)	274 (78.3)	270 (84.1)	305 (86.2)	302 (83.0)	**0.042**
Comorbidities – *n* (%)					
Diabetes Mellitus	78 (22.3)	62 (19.3)	50 (14.1)	49 (13.5)	**0.004**
Sleep Apnea	73 (20.9)	53 (16.5)	62 (17.5)	49 (13.5)	0.071
COPD	16 (4.6)	17 (5.3)	17 (4.8)	8 (2.2)	0.168
Asthma	41 (11.7)	33 (10.3)	41 (11.6)	39 (10.7)	0.921
Liver Disease	6 (1.7)	5 (1.6)	4 (1.1)	2 (0.5)	0.496
AFIB	36 (10.3)	26 (8.1)	21 (5.9)	21 (5.8)	0.075
CHF	12 (3.4)	8 (2.5)	5 (1.4)	6 (1.6)	0.256
CAD	39 (11.1)	35 (10.9)	41 (11.6)	38 (10.4)	0.969
ESRD or CKD	29 (8.3)	20 (6.2)	24 (6.8)	18 (4.9)	0.342
GERD	119 (34.0)	112 (34.9)	123 (34.7)	94 (25.8)	**0.024**
Anxiety or Depression	97 (27.7)	75 (23.4)	89 (25.1)	56 (15.4)	**0.001**
Primary HTN	205 (58.6)	187 (58.3)	193 (54.5)	200 (54.9)	0.586
Surgery Information – *n* (%)					
ASA ≥ 3	188 (53.7)	126 (39.3)	121 (34.2)	106 (29.1)	**< 0.001**
Spinal Anesthesia	192 (54.9)	204 (63.6)	238 (67.2)	246 (67.6)	**0.001**
THA	197 (56.3)	130 (40.5)	122 (34.5)	134 (36.8)	**< 0.001**

Postoperative outcomes were then stratified in terms of preoperative LEFS quartiles. Statistically significant differences in outcomes were observed across all LEFS quartiles. All statistically significant relationships showed trends toward higher levels of preoperative function being associated with superior hospital outcomes, including shorter LOS, higher rates of same day discharge, higher rates of independent ambulation, higher AM-PAC 6-Clicks mobility and activity scores, and higher rates of discharge home. A positive association between preoperative LEFS and postoperative LEFS was observed, however, less improvement from preoperative to postoperative LEFS was observed in patients with higher preoperative levels of function (Table 3).

**Table 3:** Postoperative outcomes by LEFS quartile

Variable	LEFS quartile 1 (≤ 27) *n* = 350	LEFS quartile 2 (28 to 35) *n* = 321	LEFS quartile 3 (36 to 44) *n* = 354	LEFS quartile 4 (> 44) *n* = 364	*P*-Value
LOS – avg. days ± SD	1.7 ± 1.1	1.4 ± 0.9	1.3 ± 0.8	1.2 ± 1.0	**< 0.001**
LOS – avg. hours ± SD	46.3 ± 26.5	38.6 ± 22.1	37.5 ± 19.9	35.4 ± 22.9	**< 0.001**
Same Day Discharge – *n* (%)	6 (1.7)	21 (6.5)	19 (5.4)	31 (8.5)	**0.001**
Independent Ambulation – *n* (%)^a^	173 (93.0)	199 (99.0)	216 (98.6)	203 (99.5)	**< 0.001**
Last Six Clicks Mobility Score^b^	18.2 ± 3.1	19.0 ± 2.8	19.1 ± 3.0	19.4 ± 2.9	**< 0.001**
Last Six Clicks Activity Score^c^	21.8 ± 2.7	22.4 ± 1.9	22.9 ± 1.6	22.7 ± 1.7	**< 0.001**
Discharged Home – *n* (%)	290 (82.9)	299 (93.1)	334 (94.4)	346 (95.1)	**< 0.001**
Postoperative LEFS – avg. ± SD^d^	47.0 ± 14.9	52.1 ± 12.0	52.8 ± 11.5	57.0 ± 9.5	**< 0.001**
Δ Pre- to Postoperative LEFS – avg. ± SD^e^	26.4 ± 15.4	20.8 ± 11.8	13.3 ± 11.9	3.1 ± 11.7	**< 0.001**

*P* Values < 0.05 in bold

^a^*n* = 810

^b^*n* = 976

^c^*n* = 420

^d^*n* = 490

^e^*n* = 488

To confirm the independent relationship between preoperative LEFS and postoperative outcomes, multivariate regression models were established to control for significant differences between quartiles. The control variables used included BMI, gender, race, ASA score, whether spinal anesthesia was used and the procedure performed. After adjusting for these factors, statistically significant differences in all postoperative outcomes were observed across LEFS quartiles. After risk adjustment, a higher preoperative LEFS was confirmed to be associated with all the previously described trends. Increasing LEFS quartiles were associated with reduced LOS (*β* [days] = − 0.113, *P* < 0.001; *β* [hours] = − 2.743, *P* < 0.001) and increased odds of same day discharge (OR = 1.392, *P* = 0.005). During the hospitalization, increased LEFS quartiles were associated with increased odds of independent ambulation (OR = 2.220, *P* = 0.007), increased 6-Clicks mobility and activity scores (*β* [mobility] = 0.360, *P* < 0.001; *β* [activity] = 0.333, *P* < 0.001), and increased odds of discharge home (OR = 1.554, *P* < 0.001). Postoperatively, increased LEFS quartiles were associated with higher postoperative LEFS scores (*β* = 3.016, *P* < 0.001) and decreased change in LEFS from the pre- to postoperative period (*β* = − 7.616, *P* < 0.001) (Table 4). Finally, we evaluated the relationship between the various hospital outcomes and postoperative LEFS. Statistically significant relationships with postoperative LEFS were observed for hospital LOS (*β* [days] = − 2.070, *P* = 0.001; *β* [hours] = − 0.087, *P* = 0.001), 6-Clicks mobility score (*β* = 0.756, *P* = 0.002), and home discharge (*β* = 6.923, *P* < 0.001), but not for same day discharge, independent ambulation, or 6-Clicks activity score (Table 5).

**Table 4:** Risk adjusted outcomes by LEFS quartile

Variable	Odds Ratio (*β* if denoted)	95% CI	*P*-Value
LOS Days - *β*	−0.113	− 0.158 to − 0.068	**< 0.001**
LOS Hours - *β*	−2.743	−3.833 to −1.652	**< 0.001**
Same Day Discharge	1.392	1.105–1.755	**0.005**
Independent Ambulation	2.220	1.239–3.979	**0.007**
Last Six Clicks Mobility Score - *β*	0.360	0.178–0.543	**< 0.001**
Last Six Clicks Activity Score - *β*	0.333	0.168–0.498	**< 0.001**
Discharged Home	1.554	1.281–1.885	**< 0.001**
Postoperative LEFS - *β*	3.016	2.018–4.013	**< 0.001**
Δ Pre to Postoperative LEFS - *β*	−7.616	−8.661 to −6.570	**<.001**

**Table 5:** Simple linear regression evaluating hospital outcomes as predictors of postoperative LEFS

Variable	Unadjusted *β*	95% CI	*P*-Value
LOS Days	−2.070	−3.284 to −0.856	**0.001**
LOS Hours	−0.087	− 0.137 to − 0.036	**0.001**
Same Day Discharge	1.720	−4.568 to 8.007	0.591
Independent Ambulation	8.253	−2.898 to 19.403	0.146
Last Six Clicks Mobility Score	0.756	0.284 to 1.229	**0.002**
Last Six Clicks Activity Score	0.907	−0.224 to 2.038	0.115
Discharged Home	6.923	3.140 to 10.705	**< 0.001**

*P* values < 0.05 are in bold

*LEFS* Lower Extremity Function Scale, *LOS* length of stay

## Discussion

The results of this study suggest that preoperative levels of physical function, as measured by the LEFS, are independently associated with postoperative outcomes in patients undergoing total joint arthroplasty. Patients with a higher preoperative LEFS can be expected to have a shorter LOS, are more likely to be discharged on the day of surgery, more likely to be discharged home, and demonstrate higher levels of physical function in the inpatient setting. Despite the routine use of LEFS for orthopedic patients in physical therapy clinics, the scale has not been previously used as a predictor of outcomes following TJA. Our study is the first to propose that this instrument be used as a predictive tool for the joint arthroplasty patient population which could prove impactful in developing individual treatment plans preoperatively. To our knowledge, this is the first study aimed to examine the use of LEFS to better allocate perioperative resources to optimize outcomes for the joint arthroplasty patient population.

While the relationship between preoperative LEFS scores and outcomes has not been previously described, multiple other studies have evaluated the relationship between comorbidities and outcomes of TJA [20–24]. Fisher *et al* [20] analyzed 1 year postoperative outcomes for 1024 primary total knee arthroplasty patients to identify common comorbidities among 71 patients who had a poor result either due to persistent stiffness or pain. Patient factors including demographics and comorbidity burden in the group who suffered from complications were compared with a matched control group of 148 non-painful or stiff TKA patients by using logistic regression. Female sex, higher body mass index, previous knee surgery, patients with disability, diabetes mellitus, pulmonary disease, and depression were independently associated with a stiff or painful outcome after TKA. Jain *et al* [21] also utilized multivariate logistic regressions to determine the impact of comorbidities on THA and TKA outcomes. In contrast to the previously discussed studies, the researchers adjusted for additional possible confounding variables such as age, race, household income, gender, and hospital volume during statistical analysis to isolate the influence of comorbidity burden. Hypertension, diabetes, and obesity were independent predictors of increased postoperative complications and non-home discharge in multivariable models. In addition, a combination of multiple comorbidities was also associated with increased odds of postoperative complications in hip and knee arthroplasty patients. In addition to these studies evaluating the relationship between comorbidities and complications, the influence of comorbidity burden on postoperative physical function has been previously described. Hilton e*t al* [22] performed a retrospective evaluation of primary TKA patients completing the WOMAC and SF-36 instruments. Comorbidity burden was documented via the validated Charlson comorbidity index score, an Arthroplasty Comorbidity Severity Index Score (including medical and musculoskeletal indices), and TKA-related index subscales. Increasing Charlson Index score and novel Arthroplasty Comorbidity Severity Index scores were associated with worsening physical function and painful outcomes post-TKA. Despite a body of evidence linking comorbidities and outcomes of TJA, no study to date has examined the relationship between the specific elements of physical function measured by the LEFS and outcomes.

A variety of other physical function assessments have been proposed as possible outcome predicting tools in recent studies [25–27]. Gandhi *et al* [25] and Lingard* et al* [26] evaluated the WOMAC and SF-36 as preoperative predictors of postoperative outcomes after TKA. Gandhi *et al* [25] concluded that older age, greater comorbidity burden, and poorer mental health marked by the results of the assessments described above were negative prognostic factors for the functional outcome. Lingard *et al* [26] analyzed 860 TKA patients and employed hierarchical regression models to identify the relationship between preoperative physical function metrics and surgical outcomes. Low preoperative function scores, greater comorbidity burden, and a low SF-36 mental health score were predictive of worse postoperative scores on the pain and function domains of the WOMAC and the physical function domain of SF-36 measured 1 and 2 year(s) after TKA. Weber *et al* [27] compared the predictive ability of similar presurgical measures for 140 primary THA patients. The HOOS questionnaire had the highest predictive power compared to other preoperative measures, including WOMAC, SF-36, and EQ-5D. In ROC-curve analysis, patients who did not have a positive response to THA (reduced pain and improved physical function) were identified, with a sensitivity of 91.7% and specificity of 68.9% using a cutoff value of 40.3 on preoperative HOOS. The authors concluded that patients with a HOOS score higher than 40.3 had the highest probability of a positive pain and function response after surgery. The rising popularity of TJA balanced with limited clinical resources highlights the need to develop easy predictive tools that can be incorporated into the preoperative treatment planning and patient counseling stage of care [2, 28, 29]. Based on the significant relationships observed between LEFS scores and postoperative outcomes, we suggest the instrument holds promise for its integration into predictive models that utilize multiple measures of function. Given its common use in physical therapy practice, the measure could be incorporated without imposing additional burden on patient-reported outcome data collection in busy orthopedic practices.

The validated and patient-reported nature of LEFS allows for the scale to be easily incorporated into clinical practice to shape individual treatment plans. The majority of hip and knee arthroplasty patients complete 6–8 weeks of clinic-based rehabilitation post-discharge [30]. In recent years, home-based rehabilitation has emerged as a potential cost-effective alternative to skilled nursing facilities that can produce similar functional outcomes while allowing patients to stay in the comfort of their own home [31]. The LEFS could be used to preoperatively guide patients into either clinic- or home-based postoperative rehabilitation plans or to identify patients that would benefit from several preoperative physical therapy sessions. Previous research at our institution demonstrated that home-based outpatient preoperative rehabilitation is effective at reducing postoperative discharge to skilled nursing facilities [32]. In addition, the recent removal of TKA from the Centers for Medicare and Medicaid Services in-patient only list has expanded the use of outpatient surgery centers for this patient population [33]. Utilizing the LEFS as a predictive tool could allow clinicians to appropriately counsel patients about appropriate sites of surgery and discharge disposition based on the probability of potential functional deficit which is of particular importance given the recent emphasis on transitioning more THA and TKA patients to outpatient surgery centers. Based on the results of this study, a series of quality improvement initiatives using the preoperative LEFS to guide clinical decision making, resource allocation, and patient counseling are planned. These include: (a) incorporating the LEFS into preoperative site selection algorithms to offer high-LEFS patients the opportunity to have ambulatory surgery if appropriate; (b) recommending additional preoperative rehabilitation to low-LEFS patients to potentially avoid the need for discharge to SNF; (c) targeting low-LEFS patients as priority for additional inpatient therapy to improve ambulation, activity, and mobility; (d) using the preoperative LEFS to counsel patients on the potential functional improvements they can expect postoperatively, given the reduced functional improvement observed in high preoperative LEFS patients. Future evaluations of these interventions will be performed to assess the clinical efficacy of the LEFS in practice.

The present study has several limitations. This single-center observational study might have selection bias. Furthermore, there might be additional unaccounted-for confounding variables that could impact the results of the study, including but not limited to, postoperative medical complications, and complex social determinants. To control for some of these extraneous variables, our analysis incorporated statistical techniques to control for demographic factors, comorbidities, and surgery details. However, it was still possible that other confounding factors artificially impacted the study findings. Additionally, we were unable to test the predictive value of the LEFS against other legacy PROMs, such as the WOMAC, SF-36, or EQ-5D, as these are not routinely collected in our practice. Finally, evaluation of postoperative LEFS values carries multiple limitations. The primary limitation of this measure is the variability when the last LEFS was captured over the 1 year postoperative period, as this is likely to increase as patients complete rehabilitation over time. In our study, the average time to postoperative LEFS was 110 days, but ranged from 60 to 361 days. A second limitation of this measure is its variable association with the perioperative outcome measures assessed. While the preoperative LEFS demonstrated significant relationships with all outcomes assessed, only hospital LOS, 6-Clicks mobility scores, and home discharge were associated with increased postoperative LEFS. This highlights the challenge of predicting longer term function based on hospital course, but further strengthens our assertion of the preoperative LEFS’ predictive utility given its ability to predict both hospital outcomes and postoperative function. Despite these limitations, the current study adds to previous literature by proposing a novel approach to predicting outcomes for TJA patients using a commonly used measure of functional status.

## Conclusion

Patients with a higher preoperative LEFS can be expected to have a shorter LOS, are more likely to be discharged on the day of surgery, more likely to be discharged home, and demonstrate higher levels of physical function in the inpatient setting. These findings suggest that LEFS may be a useful tool for aiding clinical resource allocation decisions, and incorporation of the measure into existing predictive models may improve their accuracy.

## Supplementary Information

**Additional file 1.**

## Data Availability

The datasets generated and/or analyzed during the current study are not publicly available due to institutional guidelines to protect patient privacy but are available from the corresponding author on reasonable request.

## Abbreviations

LEFS: Lower extremity functional scale
TJA: Total joint arthroplasty
TKA: Total knee arthroplasty
THA: Total hip arthroplasty
ASA: American Society of Anesthesiologists
LOS: Length of stay
OA: Osteoarthritis
WOMAC: Western Ontario and McMaster Universities Osteoarthritis Index
SF-36: Short Form 36
HOOS: Hip Disability and Osteoarthritis Outcome Score
EQ-5D: EuroQol 5D
PT: Physical therapy
CI: Confidence interval
OR: Odds ratio
MDC90: Minimal clinical detectable change at 90% confidence interval
ACB: Adductor canal block
ICD-10: International Classification of Disease 10th Edition
AM-PAC: Activity Measure for Post-Acute Care
ANOVA: Analysis of variance
HTN: Hypertension
GERD: Gastroesophageal reflux disease
BMI: Body mass index
SNF: Skilled nursing facility
