# Supplementary material for: The role of the lower extremity functional scale in predicting surgical outcomes for total joint arthroplasty patients
Source: Arthroplasty. 2022 Feb 1;4:3. doi: 10.1186/s42836-021-00106-3 (PMC8805277; doi:10.1186/s42836-021-00106-3)
Supplement: Supplementary file 1 — Additional file 1. [file 42836_2021_106_MOESM1_ESM.docx]

Additional file 1. The Lower Extremity Functional Scale (LEFS) instrument

# Instructions

We are interested in knowing whether you are having any difficulty at all with the activities listed below **because of your lower limb problem** for which you are currently seeking attention. Please provide an answer for **each** activity.

**Today, *do you* or *would you* have any difficulty at all with:**

| **Activities** | **Extreme difficulty or unable to perform activity** | **Quite a bit of difficulty** | **Moderate difficulty** | **A little bit of difficulty** | **No difficulty** |
| --- | --- | --- | --- | --- | --- |
| 1. Any of your usual work, housework or school activities. | 0 | 1 | 2 | 3 | 4 |
| 2. Your usual hobbies, recreational or sporting activities. | 0 | 1 | 2 | 3 | 4 |
| 3. Getting into or out of the bath. | 0 | 1 | 2 | 3 | 4 |
| 4. Walking between rooms. | 0 | 1 | 2 | 3 | 4 |
| 5. Putting on your shoes or socks. | 0 | 1 | 2 | 3 | 4 |
| 6. Squatting. | 0 | 1 | 2 | 3 | 4 |
| 7. Lifting an object, like a bag of groceries from the floor. | 0 | 1 | 2 | 3 | 4 |
| 8. Performing light activities around your home. | 0 | 1 | 2 | 3 | 4 |
| 9. Performing heavy activities around your home. | 0 | 1 | 2 | 3 | 4 |
| 10. Getting into or out of a car. | 0 | 1 | 2 | 3 | 4 |
| 11. Walking 2 blocks. | 0 | 1 | 2 | 3 | 4 |
| 12. Walking a mile. | 0 | 1 | 2 | 3 | 4 |
| 13. Going up or down 10 stairs (about 1 flight of stairs). | 0 | 1 | 2 | 3 | 4 |
| 14. Standing for 1 hour. | 0 | 1 | 2 | 3 | 4 |
| 15. Sitting for 1 hour. | 0 | 1 | 2 | 3 | 4 |
| 16. Running on even ground. | 0 | 1 | 2 | 3 | 4 |
| 17. Running on uneven ground. | 0 | 1 | 2 | 3 | 4 |
| 18. Making sharp turns while running fast. | 0 | 1 | 2 | 3 | 4 |
| 19. Hopping. | 0 | 1 | 2 | 3 | 4 |
| 20. Rolling over in bed. | 0 | 1 | 2 | 3 | 4 |
| **Column Totals:** | 0 | 1 | 2 | 3 | 4 |
